# Maltol, a Food Flavoring Agent, Attenuates Acute Alcohol-Induced Oxidative Damage in Mice

**DOI:** 10.3390/nu7010682

**Published:** 2015-01-20

**Authors:** Ye Han, Qi Xu, Jiang-ning Hu, Xin-yue Han, Wei Li, Li-chun Zhao

**Affiliations:** 1 College of Chinese Medicinal Materials, Jilin Agricultural University, Changchun 130118, China; E-Mails: hanye@jlau.edu.cn (Y.H.); xuqi@jlau.edu.cn (Q.X.); hanxinyue@jlau.edu.cn (X.H.); 2 State Key Laboratory of Food Science and Technology, Institute for Advanced Study, Nanchang University, Nanchang 330047, China; E-Mail: hujiangning@ncu.edu.cn; 3 The Affiliated Ruikang Hospital, Guangxi University of Chinese Medicine, Nanning 530011, China

**Keywords:** maltol, red ginseng, alcohol-induced liver injury, oxidative stress

## Abstract

The purpose of this study was to evaluate the hepatoprotective effect of maltol, a food-flavoring agent, on alcohol-induced acute oxidative damage in mice. Maltol used in this study was isolated from red ginseng (*Panax ginseng* C.A Meyer) and analyzed by high performance liquid chromatography (HPLC) and mass spectrometry. For hepatoprotective activity *in vivo*, pretreatment with maltol (12.5, 25 and 50 mg/kg; 15 days) drastically prevented the elevated activities of aspartate transaminase (AST), alanine transaminase (ALT), alkaline phosphatase (ALP) and triglyceride (TG) in serum and the levels of malondialdehyde (MDA), tumor necrosis factor-α (TNF-α), interleukin-1β (IL-1β) in liver tissue (*p* < 0.05). Meanwhile, the levels of hepatic antioxidant, such as catalase (CAT), superoxide dismutase (SOD), glutathione peroxidase (GSH-Px) were elevated by maltol pretreatment, compared to the alcohol group (*p* < 0.05). Histopathological examination revealed that maltol pretreatment significantly inhibited alcohol-induced hepatocyte apoptosis and fatty degeneration. Interestingly, pretreatment of maltol effectively relieved alcohol-induced oxidative damage in a dose-dependent manner. Maltol appeared to possess promising anti-oxidative and anti-inflammatory capacities. It was suggested that the hepatoprotective effect exhibited by maltol on alcohol-induced liver oxidative injury may be due to its potent antioxidant properties.

## 1. Introduction

Ethanol is a natural product that has been available for human consumption for thousands of years. It has well characterized psychophysical and mood-altering effects. It is also a common cause for the generation of reactive oxygen species (ROS), which can damage cellular lipids, proteins, and DNA leading to oxidative stress and induce liver injury [1,2,3]. Although the pathophysiological mechanism of chemical induced hepatotoxicity is not yet fully figured out, it is mostly associated with the metabolic conversion of xenobiotics into ROS, which induce oxidative stress and damage the cellular macromolecules [4]. Disturbances of the delicate balance of endogenous antioxidant defense system of the organism causes oxidative stress, which is associated with various liver disorders, therefore leading to alcoholic liver damage, non-alcoholic fatty liver disease, and drug-induced liver injury [5]. Therefore, more attention has been paid to the research and development of effective therapy for alcoholic liver disease (ALD) and agents for protecting alcohol-induced liver injury. It has been recognized that generation of free radicals and oxidative stress play a critical role in the development of ALD [6]. Recently, accumulating evidence have revealed that dietary antioxidant supplementation may contribute to keeping this balance, finally inhibiting the hepatotoxicity [7].

**Figure 1:**
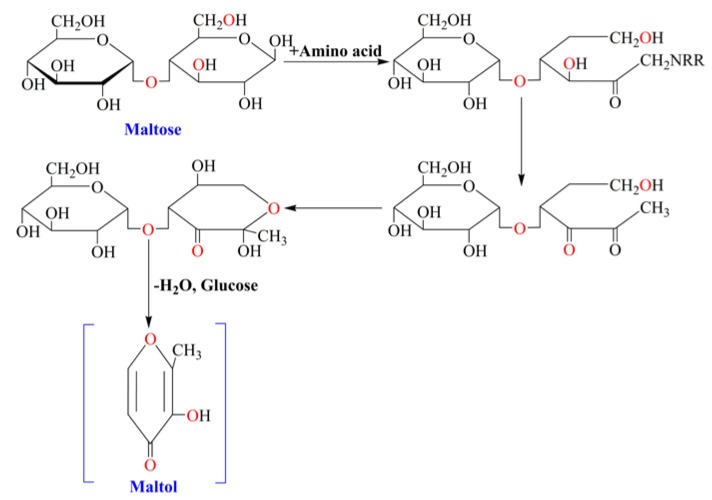
Formation pathway of maltol in maillard reaction between maltose and amino acid.

Maltol (3-hydroxy-2-methyl-4-pyrone) is one of the maillard reaction products of maltose and amino acid in heated-processed ginseng [8] (Figure 1). Maltol existed widely in nature, and it is well known as the safe and reliable flavor potentiator, food preservative and natural antioxidant in the world. Meanwhile, as one of the maillard reaction products, maltol is often found in the heat-processed food [9]. As a metal ions chelator, maltol has many practical applications in the field of catalysis, cosmetic, pharmaceutical formulations and food chemistry [10,11]. Studies showed that maltol-derived organometallic complexes have potential antitumor activity [12,13]. Furthermore, maltol can effectively protect nerve cells against oxidative damage caused by ROS in order to maintain normal physiological functions of cells [14] and inhibit diabetes-induced oxidative stress and irreversible kidney damage [15]. Meanwhile, as a growth inhibitor, maltol can be combined effectively with free radicals of body [16]. There are several lines of evidence about the antioxidant activity of maltol, and antioxidants are known to protect against ethanol-derived free radicals and may have therapeutic potential on liver injury [17,18]. As reported in previous paper, heat-processed ginseng (red ginseng) showed greater free radical scavenging activity than conventional white ginseng [19]. After heat processing, maltol content increased significantly in ginseng and showed stronger free radicals scavenging activity [20].

Currently, in addition to food industry, the research and application of maltol is also attracting more and more researchers’ attention in the medical field. Up to now, however, the protective effect of maltol on alcohol-induced liver injury has not been reported. To the best of our knowledge, this is the first report on potential preventive protection of maltol for such damage.

## 2. Experimental Section

### 2.1. Chemicals and Reagents

The red ginseng was from the roots of *Panax ginseng* C. A Meyer (Araliaceae) and identified by Professor Yi-nan Zheng, College of Chinese Medicinal Material, Jilin Agricultural University. The Maltol was isolated from the red ginseng and identified with the purity of 95.2% using HPLC method. The maltol was stored in the dryer until use. HPLC-grade acetonitrile was purchased from Fisher Scientific Co. (Hampton, NH, USA). Commercial assay kits for Aspartate transaminase (AST) microplate test kit, Alanine aminotransferase (ALT) microplate test kit, Alkaline phosphatase (ALP) microplate test kit, Triglyceride (TG) assay kit, Catalase (CAT) assay kit, Malondialdehyde (MDA) assay kit, Superoxide dismutase (SOD) assay kit and Glutathione Peroxidase (GSH-Px) assay kit were purchased from Nanjing Jiancheng Bioengineering Research Institute (Nanjing, China). Silymarin (>85.0%, UV-VIS) was separated and supplied by the Institute of Special Wild Economic Animals and Plant. Mouse tumor necrosis factor-α (TNF-α) ELISA kit and Mouse interleukin (IL)-1β ELISA kit were purchased from R & D Companies (Minneapolis, MN, USA). Other chemicals, such as alcohol were all of analytical grade from Beijing Chemical Factory.

### 2.2. Identification and Analysis of Maltol

Dried red ginseng powder of 500g was refluxed with 95% ethanol for 4 h. The extract was concentrated to dryness under vacuum at 45 °C 1.0 g of the dry extract was diluted in 10 mL water and the supernatant was filtered through a 0.45 μm nylon membrane and then injected into the HPLC for analysis.

In brief, samples were analyzed on Agilent HPLC system with UV detector. Separation was achieved on Hypersil ODS2 column (4.6 mm × 250 mm, 5 μm). The column temperature was set at 30 °C and detection wavelength was set 276 nm. The mobile phase was consisted of 8% acetonitrile with flow rate of 1.0 mL/min. The maltol were confirmed by comparing their retention time with the reference standards.

The HPLC-UV system was interfaced to the MS detector (Agilent 6100 Series Quadrupole LC/MS Systems). An electrospray ionization source was used in positive ion mode (ESI+). The capillary potential was 4.0 kV, the dry gas temperature 350 °C, the drying gas flow 12.0 L/min, and the nebulizer pressure 40 psig. Total ion chromatograms from 50 to 300 *m/z* in ESI positive modes were obtained.

### 2.3. Animals

Male ICR mice, 20–22 g, were provided by Experimental Animal Holding of Jilin University with Certificate of Quality No. of SCXK (JI) 2011–0004 (Changchun, China). Rodent laboratory chow and tap water were provided *ad libitum* and maintained under controlled conditions with 12 h light/dark cycle at 25 ± 2 °C and 60% ± 10% humidity, acclimatized for at least one week prior to use. All the procedures were in strict accordance with the China legislation on the use and care of laboratory animals.

The experiments were conducted according to the Guide for the Care and Use of Laboratory Animals (Ministry of Science and Technology of China, 2006). All experimental procedures were approved by the ethical committee for laboratory animals of Jilin Agricultural University on 15 June 2013 (Approval No. JLAU-ECLA-20130615).

### 2.4. Experimental Groups and Treatment

The animals were randomly divided into six groups (*n* = 8 per group), normal control, alcohol control, positive control (Silymarin, 50 mg/kg) and three treatment groups. Silymarin, the reference drug, is a unique flavonoid complex, including silybin, silydianin, and silychristin. It is usually used as a positive control in many investigations on hepatoprotective effect on natural compound [21,22]. The treatment groups were administered with maltol by gastric intubation for 15 consecutive days at doses of 12.5, 25 and 50 mg/kg body weight per day, respectively. Except for positive control, the normal and alcohol groups were administrated with only 0.9% saline. At day 15, normal control mice were given equal volume of water and the other mice were intragastrically administered a one-time dose of 50% ethanol (4.8 g/kg, namely 12 mL/kg) 3 h after final administration.

Then all the mice were kept fasting for 12 h, subsequently anesthetized with CO_2_. Blood samples were collected by the retrobulbar vessels and allowed to clot for 45 min at room temperature. After standing for 1 h, the serum was separated by centrifugation (1500 rpm, 10 min, and 4 °C) and stored at −20 °C for biochemical analysis.

As a reference, body weight of the animals was weighed before and after the experiment. At the end of experimental regimen, the animals in different groups were sacrificed promptly by cervical vertebra dislocation. Livers and spleens were dissected quickly, washed twice with saline, blotted dry on a filter paper, and weights were measured. At the same time, the size, appearance, and texture cut surface were recorded as well. A small piece of tissue was cut off from the same part of the left lobe of the liver in each mouse and fixed in 10% buffered formalin solution (m/v) for histopathological analysis. The remaining liver tissues were stored at −80 °C for hepatic homogenate preparation.

### 2.5. Assay for Serum Biochemical Markers

Serum was used for the spectrophotometric determination of AST, ALT, ALP and TG using commercially available diagnostic kits form Nanjing Jiancheng Institute of Biotechnology (Nanjing, China). In brief, the samples were transferred into a new 96-well plate containing substrates or buffer solution. After incubation at 37 °C, the plate was incubated for an additional time after adding color developing agent and the absorbance at 510 or 520 nm was measured. The final data are represented as U/L. TG is converted to free fatty acids and glycerol. Triacylglycerol is then oxidized to generate a product, which reacts with the probe to generate color at 570 nm.

### 2.6. Assay for Hepatic Antioxidant Activities and Oxidative Stress Marker

For the antioxidant activity assays, liver tissue was homogenated in 50 mM phosphate buffer. The resulting suspension was then centrifuged at 13,000 g for 15 min at 4 °C, and the supernatant was used for the measurement. Levels of CAT, GSH-Px and SOD in liver homogenates were measured by commercial kits according to the manufacturer’s instructions (Nanjing Jiancheng Institute of Biotechnology). The concentration of malondialdehyde (MDA) was assayed by monitoring thiobarbituric acid reactive substance formation as described by Draper and Hadley [23]. The amount of protein was measured using the Bradford assay [24].

### 2.7. Assay for Inflammatory Markers Activities

Levels of serum TNF-α and IL-1β were determined by using the ELISA kits purchased from R & D Systems (Domestic packaging, Changchun Baijin Biotechnology Ltd.) according to the protocol provided by the manufacture. Briefly, adding prepared reagent, samples and standards, antibodies labeled with enzyme, reacting 60 min at 37 °C. After adding stopping solution, measuring and calculate the OD value within 10 min.

### 2.8. Histopathological Examination

For histopathological analysis, the liver tissue (*n* = 8 per group) was fixed in 10% buffered formaldehyde for over 24 h, subsequently processed by routine paraffin embedding and sectioned for 5 μm thickness. After hematoxylin-eosin (H&E) staining, slides were observed for histopathological changes using Nikon TE 2000 fluorescence microscope (Nikon, Japan). Representative images were presented. The histopathological characters were used for assessment of histological changes of the liver, including hepatocyte degeneration or necrosis, fatty degeneration, inflammatory cell infiltration and congestion.

Assessment of hepatic steatosis, the hepatic fat accumulation was observed under light microscope and each liver section was assigned a score from Level I to IV, where Level 0 = lipid droplets in liver cells scattered, scarce and normal; Level I = hepatocytes with lipid droplets do not exceed 1/4 of the entire picture (scattered); Level II = hepatocytes with lipid droplets do not exceed 1/2 of the entire picture (scattered); Level III = hepatocytes with lipid droplets do not exceed 3/4 of the entire picture (dispersed); Level IV = lipid droplets affected almost the entire liver tissue (filled).

### 2.9. Statistical Analysis

All experiments were performed with three independent repetitions. Data were presented as means ± standard deviation (S.D). Statistical significance was determined by one-way analysis of variance (ANOVA) followed by least significance difference (LSD) multiple comparison tests using *SPSS* 16.0 software (SPSS Inc., Chicago, IL, USA). The Nonparametric Test (Ridit analyses) was used for the histological examination comparison. *p <* 0.05 was considered to be significant.

## 3. Results

### 3.1. HPLC and MS Analysis of Maltol in Red Ginseng

**Figure 2:**
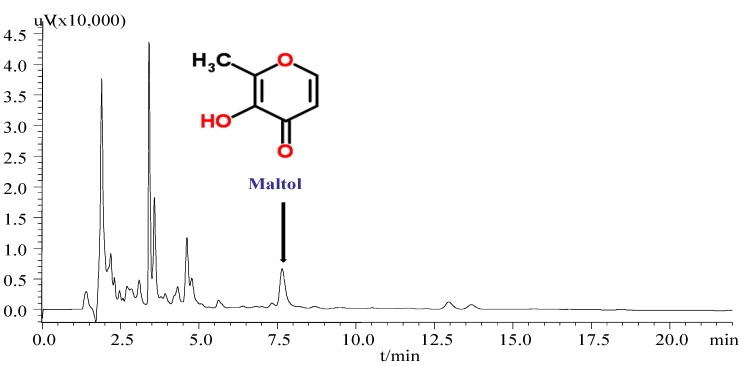
HPLC chromatogram of maltol in red ginseng.

**Figure 3:**
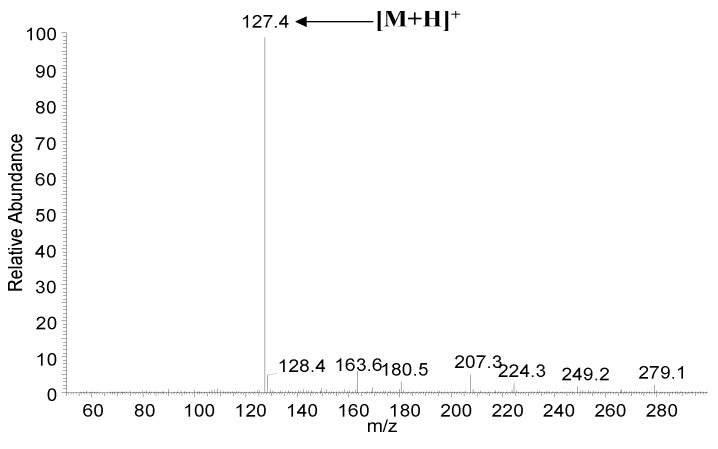
ESI-MS spectra of maltol in positive ion mode with spectrum of *m*/*z* 127.4 ([M+H]^+^ ion).

Since the maltol was derived from the heat-processed ginseng and exhibit the strong antioxidant effects [19], a simple HPLC method was used to identify the maltol content in red ginseng (Figure 2). As shown in Figure 3, an intense peak *m*/*z* 127.4 corresponding to the protonated ion [M+H]^+^ of maltol was observed. In brief, after chemical extraction and separation procedure, the structure of maltol was elucidated on the basis of UV, NMR, ESI-MS, retention times of HPLC by comparison of spectral and elemental analyses of standard maltol, and previous reported data [25].

### 3.2. Effects of Maltol on Body Weight and Organ Weight

As shown in the Table 1, the body weight is considered as a putative indicator of health. In the present study, the weight gain of mice treated with alcohol decreased in comparison with control (*p =* 0.0372), while supplementation with maltol reversed body weight loss. Alcohol-induced mice gained less weight than normal control. Liver weight in the alcohol-treated group increased significantly compared to the normal group (*p =* 0.0126), but decreased in the low dose maltol (12.5 mg/kg) groups, and significantly decreased in the Silymarin, middle and high dose (25 and 50 mg/kg) maltol-treated group. As a reference, the growth of spleen weight also showed significant decrease in the administration group, especially in the middle and high dose maltol group (*p =* 0.0211, *p =* 0.0035).

**Table 1:** Effects of maltol on body weight and organ weight in mice.

Group	Dosage (mg/kg)	Weight		Weight gain (%)	Organ Weight	
		Initial	Final		Liver (g)	Spleen (mg)
Normal		20.18 ± 1.08	31.46 ± 1.96	55.9	1.39 ± 0.12	106.96 ± 12.62
Alcohol		20.13 ± 1.08	29.53 ± 1.67	46.7 *	1.81 ± 0.22 *	129.93 ± 23.60 *
Alcohol + Silymarin	50	20.07 ± 1.31	32.26 ± 2.60	60.6	1.62 ± 0.14 ^#^	112.91 ± 16.10 ^#^
Alcohol + maltol	12.5	20.97 ± 1.01	30.19 ± 2.82	43.9	1.77 ± 0.12	117.74 ± 15.13
	25	20.32 ± 1.25	30.48 ± 2.89	50.0	1.70 ± 0.11 ^#^	103.63 ± 6.14 ^#^
	50	20.54 ± 1.05	30.73 ± 2.23	49.6	1.58 ± 0.10 ^#^	92.19 ± 3.77 ^#^

Values represent the mean ± S.D. (*n*=8); * *p <*0.05 *vs.* normal group; ^#^ *p <*0.05 *vs.* alcohol group.

### 3.3. Effect of Maltol on Serum Biochemical Markers

As showed in Table 2, the levels of ALT, AST, ALP and TG in serum are all common biomarkers of liver damage. Compared to the normal group, the activities of ALT, AST, ALP and TG were significantly elevated at 12 h following alcohol administration to mice (*p =* 0.001, 0.0034, 0.0055, 0.0097, respectively), indicated liver cell damage and the alcoholic liver injury model had been established successfully. However, maltol pretreatment for 15 days significantly decreased the levels of the serum biochemical indicators by alcohol-induced hepatic damages. Notably, administration of maltol at different doses (12.5 to 50 mg/kg) recovered the impaired liver functions to varying degrees resulting from alcohol-induced toxicity (*p <* 0.05). No significant differences were found for TG level in the low dose maltol group. The results also showed that the protective effect of high dose maltol (50 mg/kg) was equivalent to that of Silymarin (*p <* 0.05). Interestingly, treatment groups had a significant dose-dependent behavior antagonizing acute alcoholic liver injury.

### 3.4. Effect of Maltol on Hepatic Biochemical Markers Activities

**Figure 4:**
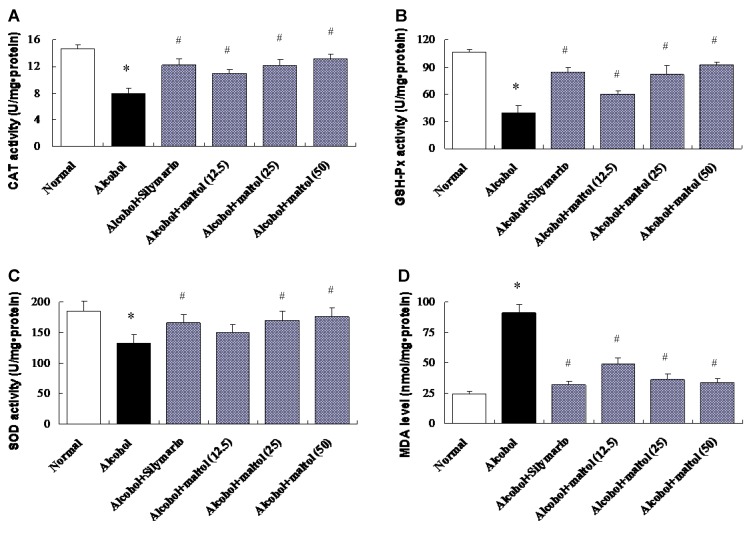
Effects of maltol on hepatic CAT (**A**), GSH-Px (**B**), SOD (**C**) and MDA (**D**) levels in alcohol-induced mice. Data represent the mean ± S.D. (*n* = 8); Significant differences were indicated by * *p<* 0.05 *vs.* normal group; ^#^ *p<* 0.05 *vs.* alcohol group; CAT, catalase; GSH-Px, glutathione peroxidase; SOD, superoxide dismutase; MDA, malondialdehyde.

The functions of liver antioxidant enzymes CAT, GSH-Px, SOD and lipid peroxidation marker MDA by the induction of alcohol to mice are summarize in the study. Compared to the control group, the hepatic antioxidant CAT, GSH-Px and SOD activities in alcohol group were decreased by 46.02%, 62.35%, and 28.49%, respectively (*p =* 0.0180, 0.0029, 0.0210, respectively). However, pretreatment with maltol for 15 days completely prevented a decrease in hepatic biochemical markers activities. A dose-dependent manner was found in all CAT, GSH-Px and SOD activities between low and high maltol administered groups respectively (*p <* 0.05), while no significant differences were observed in the low dose of SOD. On the contrary, hepatic MDA activity elevate by 71.01% in the alcohol group (*p =* 0.0013), compared to the normal group (Figure 4). The administration of maltol significantly protected against the alcohol-induced elevation of MDA activity (*p <* 0.05).

**Table 2:** Effects of maltol on serum ALT, AST, ALP and TG levels in mice.

Group	Dosage (mg/kg)	ALT (U/L)	AST (U/L)	ALP (U/L)	TG (mmoL/L)
Normal		21.26 ± 2.67	12.84 ± 3.31	23.79 ± 1.65	0.71 ± 0.10
Alcohol		55.26 ± 5.29 *	59.29 ± 4.92 *	61.03 ± 2.74 *	1.64 ± 0.28 *
Alcohol + Silymarin	50	34.02 ± 5.44 ^#^	29.06 ± 4.58 ^#^	25.67 ± 1.15 ^#^	1.09 ± 0.19 ^#^
Alcohol + maltol	12.5	32.62 ± 4.01 ^#^	27.17 ± 4.61 ^#^	33.05 ± 0.29 ^#^	1.29 ± 0.15
	25	28.57 ± 5.66 ^#^	24.52 ± 3.02 ^#^	28.10 ± 0.88 ^#^	1.12 ± 0.23 ^#^
	50	27.50 ±4.85 ^#^	26.55 ± 4.23 ^#^	27.04 ± 2.33 ^#^	0.80 ± 0.11 ^#^

Values represent the mean ± S.D. (*n*=8); * *p <*0.05 *vs.* control group; ^#^ *p <*0.05 *vs.* alcohol group; AST, aspartate transaminase; ALT, alanine aminotransferase; ALP, alkaline phosphatase; TG, triglyceride.

### 3.5. Effect of Maltol on Hepatic Inflammatory Markers

**Figure 5:**
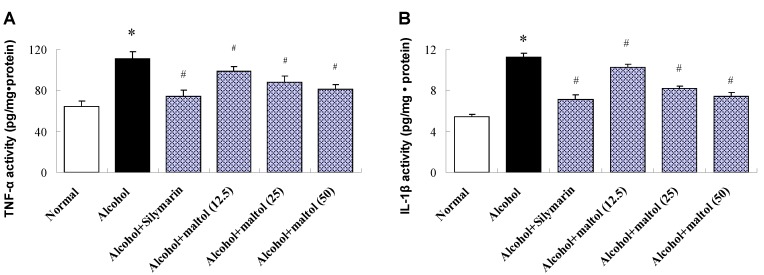
Effects of maltol on liver TNF-α (**A**) and IL-1β (**B**) in alcohol-induced mice. Data represent the mean ± S.D (*n* = 8); Significant differences were indicated by * *p <* 0.05 *vs.* normal group; ^#^ *p <* 0.05 *vs.* alcohol group; TNF-α, tumor necrosis factor-α; IL-1β, interleukin-1β.

TNF-α and IL-1β are two major inflammatory mediators implicated in inflammation. As shown in Figure 5, compared to the normal group, the results revealed considerable up-regulation of hepatic TNF-α and IL-1β after alcohol injection by 71.17%, and 83.01%, respectively (*p =* 0.0129, *p =* 0.0105). Contrarily, compared to alcohol group, pretreatment with Maltol resulted in attenuate TNF-α and IL-1β activity considerably. It is interesting to note, that it was correlated with the used dose.

### 3.6. Pathological Observations and Steatosis Grade

**Figure 6:**
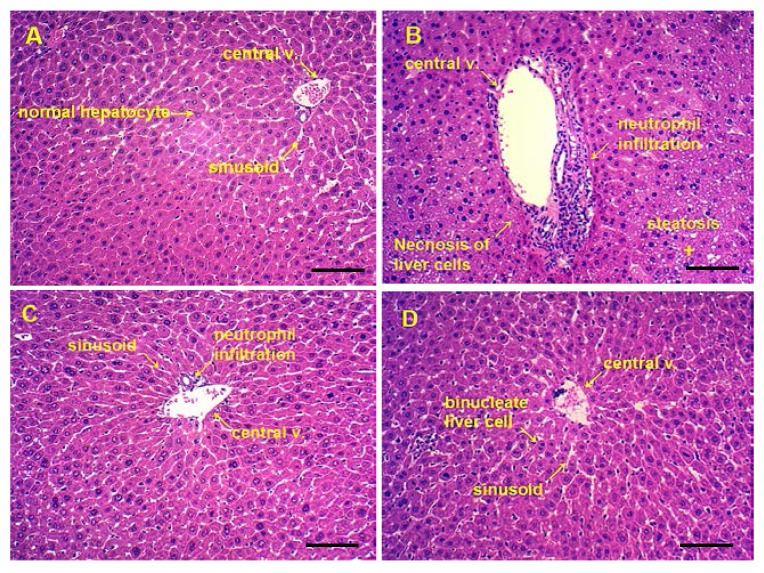
Photomicrographs of liver sections obtained from (**A**) Normal group, (**B**) Alcohol group, (**C**) Alcohol + Silymarin group (Silymarin, 50 mg/kg), and (**D**) Alcohol + maltol (50 mg/kg) (magnification, all 100×, Bar: 100 μm).

The following figures shows representative photomicrographs of livers obtained from different treatments group. The normal group had clear structure of the hepatic lobule and regular hepatic cords with central veins, the cell nucleus was normal and there was no edema, fatty degeneration or visible lesions (Figure 6A). However, in the alcohol group, typical pathological characteristics, including necrosis, inflammatory infiltration and extensive vacuolar degeneration, confirmed the successful establishment of liver injury (Figure 6B). Pretreatment of Silymarin and maltol exerted a protective effect against alcohol-induced nuclear damage, whereas Silymarin group showed only minor hepatocellular necrosis and infiltration of inflammatory cells (Figure 6C). Treatment with high dose maltol before alcohol exposure noticeably attenuated the apoptotic cells and inflammation, while almost similar to the normal group (Figure 6D). From the results, we speculate that maltol pretreatment might alleviate ethanol-induced liver damage.

As showed in Table 3, hepatic steatosis were significantly aggravated in alcohol group compared with normal group by Ridit analyses (*U* = 3.374, *p =* 0.0007). Compared with alcohol group, the degree of hepatic steatosis has made an apparent improvement in the treatment group (*U* = 2.427, *p =* 0.0152; *U* = 2.183, *p =*0.0290; *U* =2.842, *p =*0.0044; *U* =3.085, *p =*0.0020, respectively).

**Table 3:** Score result of the pathological changes in mice liver.

Groups	Dosage (mg/kg)	Steatosis Grade					Score	Ridit
		0	I	II	III	IV		
Normal		4	3	1	0	0	5	0.348
Alcohol		0	1	2	3	2	19	0.835 *
Alcohol + Silymarin	50	2	4	1	1	0	9	0.484 ^#^
Alcohol + maltol	12.5	2	3	2	1	0	13	0.520 ^#^
	25	3	3	2	0	0	9	0.425 ^#^
	50	3	3	2	0	0	7	0.389 ^#^

Code of point (*n* = 8): Level 0 calculated 0 mark; Level I calculated 1 mark; Level II calculated 2 marks; Level III calculated 3 marks; Level IV calculated 4 marks. * *p <* 0.05 *vs.* normal group; ^#^ *p <* 0.05 *vs.* alcohol group.

## 4. Discussions

Reactive oxygen species (ROS), one kind of pro-oxidants including superoxide radical, hydroxyl radical, hydrogen peroxide and nitric oxide, are constantly generated spontaneously in the biological systems during metabolism. In the normal condition, it is very important to maintain a certain level of ROS for oxidation-reduction balance and cell proliferation. Generally, ethanol can produce ROS by a plurality of pathways [26]. The alcohol mediated oxidative stress and the decline of scavenging activity of free radical is one of the main causes of hepatic damage, which is widely evidenced in rodents and humans. Simultaneously, the involvement of free radical mechanisms in the pathogenesis of ALD is demonstrated by the detection of lipid peroxidation markers in the liver and the serum of patients with alcoholism [27,28]. Most ingested ethanol is metabolized in the liver, excessive ethanol intake results in ALD, such as hepatic steatosis, inflammation, hepatitis, fibrosis and even cirrhosis [29].

Maltol, generated from heat treatment such as roasting process, is used as a food flavor-enhancing agent for many years. As reported previously, maltol exhibits good antioxidant activity *in vitro* [8,9]. In addition, the neuroprotective effect of maltol on the oxidative stress in the brain of mice was investigated [30]. However, the hepatoprotective action of maltol against liver oxidative stress was not reported so far. Hepatocyte damage is characterized by serum marker enzymes including ALT, AST, ALP and TG levels, which reflect the early biochemical and pathological changes in ALD [31,32,33]. ALT is a cytosolic enzyme mainly present in the cell cytoplasm, and AST is a mitochondrial enzyme, which is released from the liver and other organs in the body. ALP is produced in the tissues especially in the bone and liver. The elevated serum levels of this enzyme, due to defective hepatic excretion or increased production by hepatic parenchymal or duct cells, indicate hepatobiliary disease [34]. When liver cells are damaged, these enzymes leak into the bloodstream from liver tissue and produce markedly elevated serum levels [35]. In the early stage of the liver disease, TG accumulates in hepatocytes, leading to the development of fatty liver [36]. However, treatment with different doses of maltol with ethanol markedly recovered ALT, AST, ALP and TG levels to near normal control mice, suggesting that maltol may have a hepatoprotective effect against alcohol-induced hepatotoxicity. Interestingly, the hepatoprotective effect of Silymarin was consistent with other investigations on alcohol induced liver injury [22].

Current research suggests that antioxidant enzymes such as CAT, GSH-Px and SOD, which are scavengers of free radicals or ROS in the liver, are the first line of defense against oxidative injury. It also has been evidenced that CAT, GSH-Px and SOD can be inactivated by peroxides, oxygen-derived free radicals and reactive nitrogen species [37]. CAT helps to remove hydrogen peroxide become H_2_O and avoid the production of greater toxicity of hydroxyl radicals. GSH-Px plays a protective role in the low levels of oxidative stress by remove lipids and other organic peroxides effectively. While SOD gets through continuous oxidation and reduction of metal ions of transition state, catalyzing the removal of superoxide radicals [38]. Once the hepatic antioxidant activities are inactivated by alcohol-mediated ROS and radicals, the liver will be attacked [39]. In the present study, pretreatment of maltol before ethanol exposure and simultaneously with ethanol enhanced CAT, GSH-Px and SOD activities, which may be a reflection of their increased synthesis in the liver [40]. MDA is widely used as a marker of lipid peroxidation and a major parameter for the status of oxidative stress [41]. It is reported that the hepatic MDA level was increased under the enhancement of oxidative stress in a rodent model [42]. In the present paper, the results revealed that maltol could provide protective effects against alcohol-induced liver damage in terms of balancing the above enzyme levels in the liver and preventing lipid peroxide formation and blocking oxidative chain reaction.

In addition to oxidative stress, the key feature of ALD is the cytokine's metabolism. Inflammation is a protective attempt by damaged tissues to remove noxious stimuli and to treat wounds. TNF-α and IL-1β are two important inflammatory cytokines involved in ALD [43]. Inflammation is a complex phenomenon involving multiple cellular and molecular interactions which must be rigorously controlled to avoid different pathology and disorders [44]. TNF-α is a key mediator in many experimental liver injury models, which could mediate acute and chronic inflammation and infection [45,46]. It could promote cytokines generation and inflammation reaction by stimulating neutrophils and macrophages, ultimately resulting in liver cell necrosis or apoptosis [47]. In addition to promoting inflammation, causing fever outside, IL-1β can stimulate development and differentiation of the immune system [48]. Clinical research indicates that TNF-α and IL-1β levels in serum of ALD patients is closely related to acute phase response markers, liver function and clinical outcome [49]. This study shows that disposable ethanol feeding makes expression of TNF-α and IL-1β significantly increased in the liver.

Considering the protective effect of maltol on alcohol-induced liver injury in mice, the development of maltol supplementation could be useful to ameliorate liver damage for human. According to the calculated results, individual with body weight of 60 kg may consume approximately 200 mg per day, and this dose was far less than its toxicity dose.

## 5. Conclusions

In conclusion, the current investigation is the first report indicating that pretreatment of maltol is effective in the prevention of alcohol-induced hepatic damage in mice. It is speculated that the mechanism of hepatoprotective by maltol supplementation against alcohol toxicity might be due to the alleviation of oxidative stress via preventing lipid peroxidation and ameliorating hepatic antioxidant status.
